# Veinte años de la apendicectomía transumbilical en Navarra: Acabamos de empezar

**DOI:** 10.23938/ASSN.1082

**Published:** 2024-07-03

**Authors:** Julio César Moreno-Alfonso, Ada Molina-Caballero, Alberto Pérez-Martínez

**Affiliations:** 1 Servicio Navarro de Salud-Osasunbidea Hospital Universitario de Navarra Cirugía Pediátrica Pamplona Navarra España; 2 Universidad Pública de Navarra (UPNA) Escuela de Doctorado en Ciencias de la Salud Pamplona Navarra España

Sra. Editora:

La apendicitis aguda es la urgencia quirúrgica abdominal más frecuente en el ser humano, con un riesgo acumulado a lo largo de la vida de 7-9%^1^, y la población pediátrica no es la excepción.

Actualmente, el conocimiento de la etiología, fisiopatología y tratamiento de la apendicitis aguda ha aumentado considerablemente, de tal forma que el abordaje vía laparotomía -técnica de elección durante un amplio periodo de tiempo- ha pasado a un segundo plano y las técnicas de mínima invasión, fundamentalmente la laparoscopia, se han convertido en el acceso quirúrgico de elección para el tratamiento quirúrgico de esta enfermedad^2^, también en los pacientes pediátricos.

**Figura 1:**
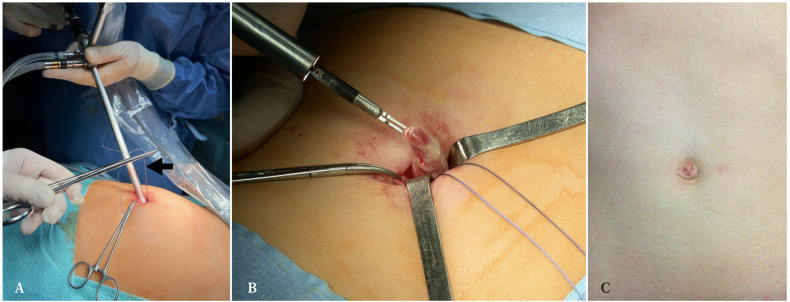
A. Introducción de la óptica de laparoscopia a través del ombligo y sutura continua en *bolsa de tabaco* rodeando la fascia umbilical para evitar la fuga del neumoperitoneo (flecha). B. Exteriorización del apéndice cecal vía umbilical. C. Aspecto de la cicatriz en el postoperatorio tardío.

Sin embargo, en Navarra nuestro equipo tomó un rumbo diferente. Sin abandonar el terreno de la cirugía mínimamente invasiva, consideramos que la apendicectomía laparoscópica convencional, con tres incisiones y un elevado coste económico, podía sustituirse por una técnica realizada con una única incisión en el ombligo. Las ventajas de esta sustitución son menores costes, menor dolor postoperatorio y mejor resultado estético, todo ello con los mismos resultados, o mejores, que las otras opciones quirúrgicas. De esta forma obtenemos la excelente visualización y beneficios perioperatorios de la cirugía mínimamente invasiva sin perder la seguridad y maniobrabilidad de la cirugía abierta, con unos resultados cosméticos difícilmente mejorables (Fig. 1).

El 11 de septiembre de 2003 introdujimos por primera vez un toracoscopio a través del ombligo de un niño de 10 años con sospecha de apendicitis aguda, comprobando el diagnóstico. Exteriorizamos el apéndice a través de la incisión umbilical y lo extirpamos sin complicaciones de forma convencional. Esta fue la primera apendicectomía transumbilical videoasistida de nuestra institución, y tal vez la primera -hasta donde tenemos conocimiento- en España. Conforme hemos adquirido experiencia, esta técnica se ha convertido en el acceso de elección para el tratamiento quirúrgico de la apendicitis aguda y un abordaje adicional en muchas otras indicaciones, como las lesiones intestinales secundarias a traumatismos, la cirugía hepatobiliar, gastrointestinal, urológica y ginecológica^3^. Veinte años después seguimos perfeccionando la técnica, enriqueciéndola y complementándola con diferentes maniobras y dispositivos, procurando extrapolar este abordaje a todas aquellas indicaciones quirúrgicas en las cuales sea posible resolver con seguridad el problema en cuestión.

A día de hoy hemos realizado más de 2.300 intervenciones quirúrgicas por esta vía, fundamentalmente apendicectomías pero también resecciones intestinales por traumatismos o malformaciones congénitas, colecistectomías y toma de biopsias, entre otras^3^^,^^4^. Un estudio publicado por nuestro grupo en 2007 con 208 pacientes operados por apendicitis aguda sirve para ilustrar los mejores resultados observados en las 162 apendicectomías por la vía transumbilical (77,9%) respecto a las 48 apendicectomías abiertas (23,1%) realizadas durante un año: significativamente menor estancia media (2,8 frente a 4,8 días), menor dolor postoperatorio y menor necesidad de analgesia, a pesar de una mayor frecuencia (no significativa) de complicaciones infecciosas (4,2 frente a 1,7%) y por último, pero no menos importante, la técnica transumbilical supuso un ahorro de aproximadamente 90.561 €/año comparado con el coste que supondría la apendicectomía laparoscópica, y unos 42.232 €/año comparado con la cirugía abierta tradicional, sin aumento de la morbimortalidad^5^.

**Figura 2:**
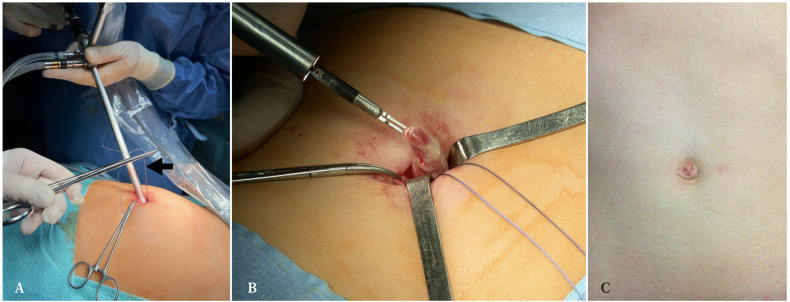
A. Guante estéril acoplado al retractor quirúrgico y trócares colocados a su través en la realización de una colecistectomía laparoscópica por puerto único transumbilical. B. Exteriorización de íleon vía umbilical en una perforación traumática para realización de resección intestinal y anastomosis latero-lateral mecánica.

Estos 20 años de cirugía transumbilical nos han enseñado varias lecciones:

- por su privilegiada localización y flexibilidad de la pared abdominal en los niños, el ombligo permite acceso casi a cualquier estructura peritoneal;
- el uso de retractores quirúrgicos permite aumentar el diámetro de la incisión umbilical y proteger el trayecto de la herida para extraer a su través diferentes órganos y culminar el procedimiento fuera de la cavidad abdominal como en la cirugía abierta (Fig. 2B);
- a su vez, al borde plástico del retractor se puede acoplar un guante u otros dispositivos prefabricados para insertar instrumentos adicionales, con o sin trócar, y realizar un procedimiento laparoscópico a través de una única incisión umbilical (Fig. 2A);
- el bloqueo anestésico ecoguiado de la vaina de los rectos es un complemento excelente a la analgesia multimodal, con buenos resultados en el control del dolor postoperatorio;
- el uso de suturas impregnadas con triclosán y adhesivos tisulares cutáneos puede aportar seguridad y simplicidad al procedimiento;
- la neumostasia suele mantenerse adecuadamente con la sutura en bolsa de tabaco; no obstante, si el cirujano lo prefiere también se pueden emplear trócares. Nosotros prescindimos de estos, porque para procedimientos cortos creemos que resulta más cómodo, económico y proporciona más libertad en los movimientos del instrumental, y
- este abordaje permite además la digitoclasia o maniobras bimanuales, lo cual resulta especialmente útil en algunos casos de apendicitis evolucionadas o latero-retrocecales.

La técnica transumbilical ha evolucionado sustancialmente; sin embargo, consideramos que aún tiene mucho que ofrecer y podría tener un futuro prometedor con la implementación de la cirugía robótica y el uso de pincería articulada que permitiría mayor maniobrabilidad y mejor triangulación por esta vía, que pueden ser algunas de sus limitaciones^6^. Actualmente, un amplio grupo de colegas nacionales e internacionales emplean la vía transumbilical en diferentes indicaciones; esperamos que con su experiencia podamos hacer crecer la técnica y sacar el máximo provecho a esta en beneficio de nuestros pacientes.

Terminamos este relato, que asumimos como simples interlocutores de una parte de la cirugía pediátrica española, sin posturas pretenciosas pero orgullosos de ser el grupo con mayor experiencia mundial en este campo de la cirugía pediátrica, recordando que el cirujano pediátrico no debería olvidar la *oración-súplica* que en 1959 escribiera Willis J. Potts al referirse a un niño afectado por una malformación grave:

“[...] *si esa criatura pudiera hablar, le pediría al cirujano en tono suplicante: Por favor, emplee la mayor delicadeza en mis minúsculos tejidos y trate de corregir la deformidad en la primera operación, deme sangre y la cantidad precisa de líquidos y electrolitos, añádale oxígeno a la anestesia y le demostraré que soy capaz de tolerar una intervención quirúrgica de enorme amplitud. Se asombrará de la rapidez de mi recuperación; de mi parte, le quedaré eternamente agradecido* [...]”.
